# Effects Of A Post-Weaning Cafeteria Diet In Young Rats: Metabolic Syndrome, Reduced Activity And Low Anxiety-Like Behaviour

**DOI:** 10.1371/journal.pone.0085049

**Published:** 2014-01-15

**Authors:** Jaume F. Lalanza, Antoni Caimari, Josep M. del Bas, Daniel Torregrosa, Igor Cigarroa, Mercè Pallàs, Lluís Capdevila, Lluís Arola, Rosa M. Escorihuela

**Affiliations:** 1 Institut de Neurociències, Departament de Psiquiatria i Medicina Legal, Universitat Autònoma de Barcelona, Barcelona, Spain; 2 Laboratori de Psicologia de l'Esport, Departament de Psicologia Bàsica, Universitat Autònoma de Barcelona, Barcelona, Spain; 3 Centre Tecnològic de Nutrició i Salut (CTNS), TECNIO, CEICS, Reus, Spain; 4 Grado de Kinesiología, sede Los Ángeles, Facultad de Salud, Universidad Santo Tomás, Chile; 5 Secció de Farmacologia i Farmacognòsia, Facultat de Farmàcia, Institut de Biomedicina (IBUB), Universitat de Barcelona, Barcelona, Spain; 6 Centros de Investigación Biomédica en Red de Enfermedades Neurodegenerativas (CIBERNED), Spain; 7 Departament de Bioquímica i Biotecnologia, Nutrigenomics Research Group, Universitat Rovira i Virgili, Tarragona, Spain; The University of Manchester, United Kingdom

## Abstract

Among adolescents, overweight, obesity and metabolic syndrome are rapidly increasing in recent years as a consequence of unhealthy palatable diets. Animal models of diet-induced obesity have been developed, but little is known about the behavioural patterns produced by the consumption of such diets. The aim of the present study was to determine the behavioural and biochemical effects of a cafeteria diet fed to juvenile male and female rats, as well as to evaluate the possible recovery from these effects by administering standard feeding during the last week of the study. Two groups of male and female rats were fed with either a standard chow diet (ST) or a cafeteria (CAF) diet from weaning and for 8 weeks. A third group of males (CAF withdrawal) was fed with the CAF diet for 7 weeks and the ST in the 8th week. Both males and females developed metabolic syndrome as a consequence of the CAF feeding, showing overweight, higher adiposity and liver weight, increased plasma levels of glucose, insulin and triglycerides, as well as insulin resistance, in comparison with their respective controls. The CAF diet reduced motor activity in all behavioural tests, enhanced exploration, reduced anxiety-like behaviour and increased social interaction; this last effect was more pronounced in females than in males. When compared to animals only fed with a CAF diet, CAF withdrawal increased anxiety in the open field, slightly decreased body weight, and completely recovered the liver weight, insulin sensitivity and the standard levels of glucose, insulin and triglycerides in plasma. In conclusion, a CAF diet fed to young animals for 8 weeks induced obesity and metabolic syndrome, and produced robust behavioural changes in young adult rats, whereas CAF withdrawal in the last week modestly increased anxiety, reversed the metabolic alterations and partially reduced overweight.

## Introduction

In recent decades, the prevalence rates of paediatric overweight and obesity have been increasing, reaching 20% or more in some countries [1],[2]. Paediatric obesity is frequently associated with cardiometabolic risk factors and severe components of the metabolic syndrome, such as impaired insulin resistance, high glucose, triglycerides and HDL-cholesterol levels, as well as high blood pressure values [3],[4].

Animal models developed to induce obesity in rodents are usually based on different forms of diet containing high fat, high sugar or palatable foods regularly consumed by humans. Among such diets, the cafeteria (CAF) diet has been shown to consistently increase body weight, induce hyperphagia and alter the metabolic factors clustered in the metabolic syndrome [5],[6]. Moreover, because the CAF diet also produces inflammation in fat and liver, it is a unique and important platform for studying obesity, as it induces the human metabolic syndrome-like phenotype more effectively than a high-fat diet (HFD) [6]. Administration of the CAF diet for 15 days to young adult male and female rats increased adiposity and decreased adiponectin production in white adipose tissue, whereas the levels of insulin in plasma, insulin sensitivity and body weight did not change in males [7]. This finding was interpreted as an early response to obesity because, in both rats and humans, a decreased production of adiponectin secreted by adipose tissue is a characteristic of obesity [8],[9] that, in turn, could contribute to insulin resistance. In another study, the CAF diet administered to female rats, from weaning to adulthood, induced obesity by increasing body and fat pad weight, resulting in increased levels of triglycerides, total cholesterol and LDL-cholesterol, as well as induced insulin resistance and reproductive deficits [10].

From a different perspective, emotions are related with eating behaviour. Consistent data suggest that negative mood induces overeating and weight gain, and that eating behaviour reduces anxiety [11]. Palatability is critical in determining food preference [12], and it appears that high levels of stress can increase the consumption of this type of food [13]. A recent study of college students has shown that the desire for and consumption of palatable food were augmented by negative emotions, such as stress, boredom or anxiety [14]. However, the direct emotional implications of eating behaviour and obesity in animal models have not been determined. Work undertaken with non-obese rats showed that low-anxiety rats had a higher preference for a palatable high-fat diet than did high-anxiety rats [15], and that a palatable HFD or chocolate partially ameliorated anxiety as well as depressed mood levels caused by adverse early life experiences [16],[17]. Yet, in contrast, another study showed that diet-induced obese rats were more anxious and aggressive than the non-obese ones [18]. In that study, animals were fed with a high-fat non-palatable diet, thereby, emphasizing food palatability as a possible factor required for reducing anxiety. High-fat diets are usually a semi-purified chow diet with an elevated calorie content that, in rodents, did not cause the noticeable hyperphagia that a palatable cafeteria diet does [6],[19].

The present experiment aimed to evaluate the behaviour of obese male and female rats fed with the CAF diet in terms of locomotor activity, exploration, anxiety-like behaviour and social interaction tests. As we were interested in studying cafeteria diet-induced obesity in young adulthood, animals were fed with the corresponding CAF or standard (ST) diets for 8 weeks starting at weaning. Our hypothesis was that, as a result of CAF feeding, anxiety-related behaviour and locomotor activity would decrease in young adult obese animals. Body weight, liver weight, retroperitoneal white adipose tissue (RWAT) weight and biochemical parameters clustered in the metabolic syndrome were evaluated to determine diet and gender dependent effects and/or interactions on these variables. It was also investigated whether or not 7 weeks of CAF diet, followed by a shift to standard chow diet during the final 8th week (CAF withdrawal) could partially reverse the negative effects of the palatable CAF diet.

## Results

### Impact Of Caf Diet On Body And Tissue Weights

**Figure 1:**
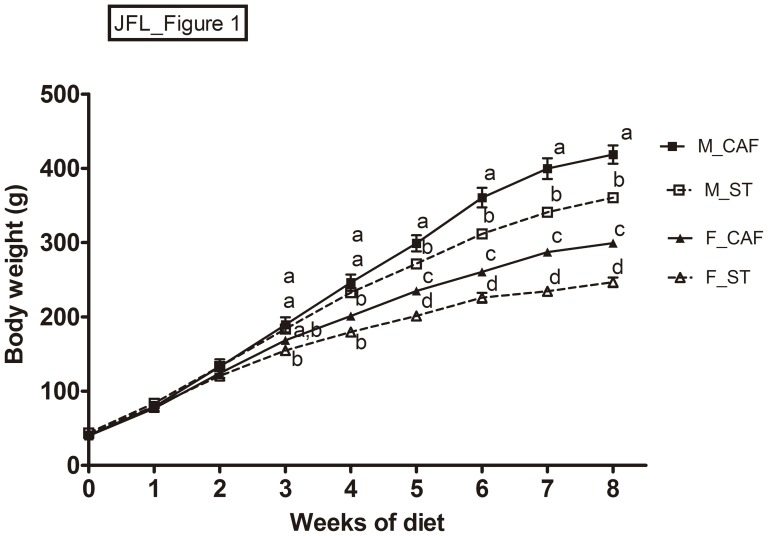
Male (M) and female (F) rats were fed from weaning (at day 21 of life, week 0) and for 8 weeks with standard chow (ST) or cafeteria diet (CAF). The data represent mean ± SEM of body weight (n = 8). The increase of body weight over the 8-week period was greater in animals fed with the CAF diet than in animals fed with the ST diet (diet x week interaction), as well as in males than females (gender x week interaction, two-way ANOVA for repeated measures). ^abcd^Mean values with unlike letters were significantly different between groups (one-way ANOVA and Tukey's post hoc comparison, *p*<0.05).

At the beginning of the experiment, body weight did not differ between male and female rats. CAF-fed males and females increased the amount of solid and fluid intake compared to ST-fed animals, with a consequent increase in energy and macronutrient consumption (see Table S1). Body weight increased progressively over weeks, and both male and female CAF-fed animals were significantly overweight from week 5 of the diet onwards, as compared to the corresponding ST-fed rats of the same sex (Figure 1). At the end of the study (8th week), CAF-fed males and females had become overweight by 16.3% and 21.1%, respectively, in comparison with their respective ST-fed controls. Relative liver weight (%) and relative RWAT weight (%) were significantly increased in CAF-fed animals compared with the ST-fed groups (Table 1). This latter effect on RWAT was more pronounced in females than in males (Table 1).

**Table 1:** Biometric and plasma parameters of male and female rats fed with a standard chow diet (ST) or a cafeteria diet (CAF) + standard chow diet.

	M_ST	M_CAF	F_ST	F_CAF		M_ABS
**Biometric parameters**						
Body weight gain (g)	316±6^A a^	378±11^B b^	204±7^C^	259±4^D^	*D, G*	350±9^b^
Liver weight (%)*	2.8±0.1^A a^	3.1±0.1^B b^	2.6±0.1^A^	2.7±0.1^A^	*D, G*	2.8±0.1^a^
RWAT weight (%)*	0.80±0.05^A a^	1.9±0.3^B b^	0.82±0.05^A^	2.9±0.1^C^	*D, G, DxG*	1.7±0.2^b^
**Plasma parameters**						
Glucose (mmol/L)	5.6±0.2^A a^	6.8±0.3^B b^	5.6±0.1^A^	6.8±0.3^B^	*D*	6.0±0.2^ab^
Insulin (mmol/L)	7.8±1.1^A a^	26.4±7.0^B b^	4.8±0.6^A^	21.2±4.4^B^	*D*	8.7±1.4^a^
HOMA-IR	2.0±0.3^A a^	8.6±2.6^B b^	1.2±0.2^A^	6.6±1.6^B^	*D*	2.3±0.4^a^
Triglycerides (mmol/L)	0.70±0.03^A a^	1.4±0.05^B b^	0.70±0.04^A^	1.0±0.05^C^	*D, G, DxG*	0.80±0.1^a^
Total cholesterol (mmol/L)	3.1±0.2^A^	2.9±0.1^A^	3.7±0.2^B^	4.7±0.1^C^	*D, G, DxG*	3.1±0.2
Free fatty acids (mmol/L)	0.70±0.02^AB^	0.64±0.04^A^	0.80±0.02^B^	0.68±0.04^AB^	*D*	0.61±0.03

Legend Table 1. Male (M) and female (F) rats were fed from weaning (at day 21 of life) and for 8 weeks with a ST (M_ST and F_ST groups) or a CAF diet (M_CAF and F_CAF groups). An extra group of males were fed with the CAF diet for 7 weeks and with the ST diet only for the final week of the study (M_ABS group). The data are given as the mean ± SEM (n = 7–8). *Relative liver weight and relative RWAT weight were calculated following the formula (100* tissue weight/body weight) and expressed as a percentage of the total body weight. The statistical comparison between males and females fed with a ST (M_ST and F_ST groups) or a CAF diet (M_CAF and F_CAF groups) was performed by two- and one-way ANOVA. *D*: the effect of the type of diet. *G*: the effect of the gender (two-way ANOVA, p<0.05). ^ABC^ Mean values within a row with unlike capital letters were significantly different between groups (one-way ANOVA and Tukey's post hoc comparison, p<0.05). In males (M_ST, M_CAF and M_ABS groups), ^ab^ mean values within a row with unlike small letters indicate where there are significant differences between groups (one-way ANOVA and Tukey's post hoc comparison, p<0.05).

### Metabolic Consequences Of Caf Feeding

As shown in Table 1, 8 weeks of CAF feeding significantly increased the plasma glucose and insulin levels in both males and females. Consequently, the homeostatic model assessment for insulin resistance (HOMA-IR) was higher in these animals, indicating a decrease in insulin sensitivity as a consequence of the CAF diet intake. Both male and female CAF-fed animals showed increased plasma triglyceride levels, with this effect being more evident in males than in females (Table 1). The plasma cholesterol levels were higher in females than in males, and CAF feeding only raised this parameter in females (Table 1). In both male and female rats, there were no changes in free fatty acid levels in response to CAF feeding (Table 1).

### Caf Feeding Increased Exploration

**Figure 2:**
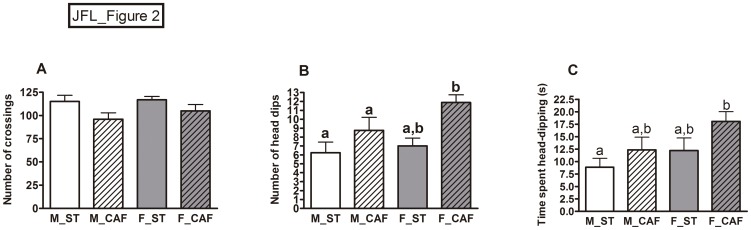
Response to novelty in the hole board. The number of crossings (A), number of head dips (B) and time spent head dipping (C) during the trial was evaluated in male (M) and female (F) rats fed from weaning (at day 21 of life) and for 8 weeks with standard chow (ST) or the cafeteria diet (CAF). The data are given as the mean ± SEM (n = 8). Two-way ANOVAs revealed that the CAF diet decreased the number of crossings (*p*<0.05) and increased head-dipping behaviour (number: *p*<0.01; time: *p*<0.05). Females spent moderately more time head dipping than males (*p* = 0.051, gender effect). ^ab^Mean values with unlike letters were significantly different between groups (one-way ANOVA and Tukey's post hoc comparison, *p*<0.05).

Animals were tested using the hole-board apparatus to evaluate motor activity by the number of crossings (the floor was divided into 16 squares) and exploratory behaviour through the holes in the floor (head-dipping behaviour). More exploration (frequency and duration of head dips) is indicative of high curiosity and interest in novelty-seeking behaviour [20]. Overall, CAF feeding reduced the number of crossings (Figure 2A), but increased both the number of head dips and the time spent head dipping (Figures 2B and 2C), thereby indicating reduced motor activity and increased exploratory behaviour in the CAF-fed animals compared to the ST-fed animals. Females showed a tendency to explore more than males (gender effect: number of head dips: *p* = 0.098; time spent head dipping: *p* = 0.051) (Figures 2B and 2C).

### Caf Feeding Decreased Activity And Anxiety

To evaluate anxiety-like behaviour, the animals were tested in the elevated plus maze (EPM) consisting of two open arms and two enclosed arms with walls alternatively positioned forming the shape of a plus sign. A high amount of time and number of entries into the open arms is considered to indicate low anxiety [21], while the distance travelled in the enclosed arms is considered to be a measure of locomotion [22].

**Figure 3:**
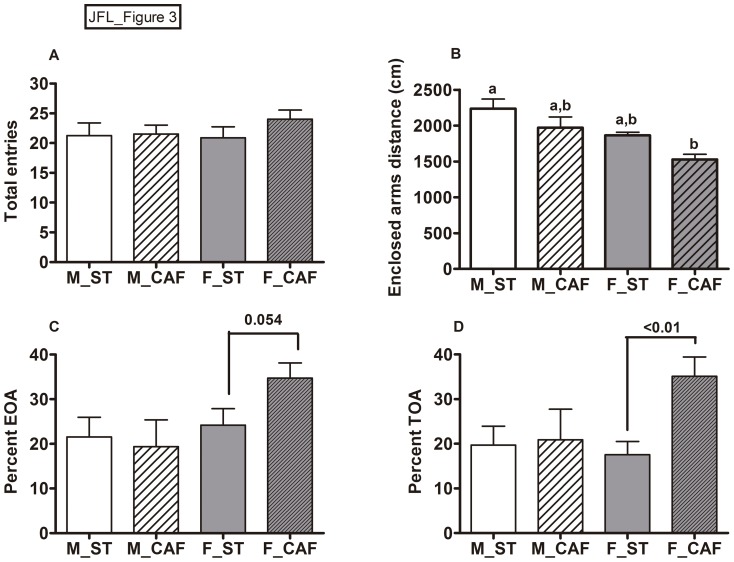
Behaviour in the elevated plus maze test. The number of total entries (A), distance travelled in the enclosed arms (B), percentage of entries into the open arms (C) and percentage of time spent in the open arms (D) during the trial was evaluated in male (M) and female (F) rats fed from weaning (at day 21 of life) and for 8 weeks with standard chow (ST) or the cafeteria diet (CAF). The data are given as the mean ± SEM (n = 8). Two-way ANOVAs revealed that the CAF diet decreased the distance travelled in the enclosed arms (diet effect: *p*<0.05) and slightly increased the percentage of TOA (diet effect: *p* = 0.061). Females travelled shorter distances than males in the enclosed arms (gender effect: *p*<0.01) and moderately increased the percentage of EOA (gender effect: *p* = 0.055). ^ab^Mean values with unlike letters were significantly different between groups (one-way ANOVA and Tukey's post hoc comparison, *p*<0.05). The significance of a Student's t-test between the groups indicated is shown in C) and D), revealing that the F_CAF group increased behaviour in the open arms compared to the F_ST group.

The intake of the CAF diet reduced the travelled distance in the enclosed arms in both males and females (Figure 3B), while in females, it increased the behaviour in the open arms. CAF-fed females almost doubled the percentage of time spent in the open arms (Figure 3D), and residually increased the percentage of entries in the open arms compared with ST-fed females (Figure 3C).

Overall, females were less active and slightly less anxious than males, as they travelled shorter distances in the enclosed arms (Figure 3B), and also slightly increased the percentage of entries in the open arms in comparison with the males (Figure 3C).

**Figure 4:**
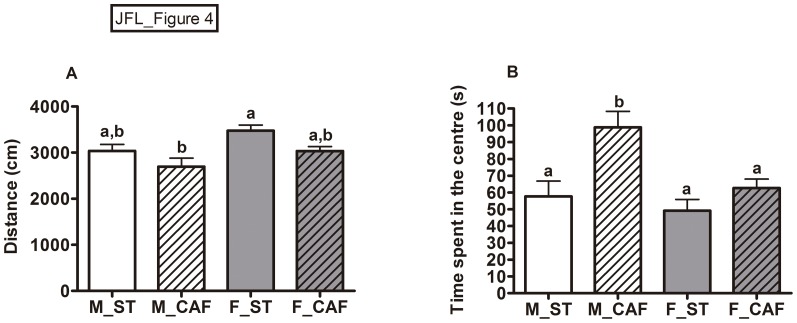
Open field test. The behaviour of the rats was measured during the 5-min session. Panels A and B show the total distance and the time spent in the central area of the apparatus. The data are given as the mean ± SEM (n = 8). The CAF diet decreased the distance travelled (*p*<0.01) and increased time in the centre (*p*<0.01; diet effect in the two-way ANOVA). Females were more active (*p*<0.01) and spent less time in the centre (*p*<0.01) compared to males (gender effect in the two-way ANOVA). ^ab^ Mean values with unlike letters were significantly different between groups (one-way ANOVA and Tukey's post hoc comparison, *p*<0.05).

The analyses of activity and anxiety variables measured in the open field were consistent with the results of the EPM. Again, CAF feeding had reduced locomotor activity since CAF-fed male and female rats showed an overall reduction in the distance travelled in the test arena (Figure 4A). Moreover, as CAF-fed animals spent significantly more time in the central area of the open field, they again displayed reduced anxiety-like behaviour, which was lower than that of the animals fed with ST chow (Figures 4B). Regarding gender behavioural patterns, overall females were more active (Figures 4A), and spent less time in the central area of the open field than males (Figure 4B).

### Caf Feeding Increased Social Behaviour

**Figure 5:**
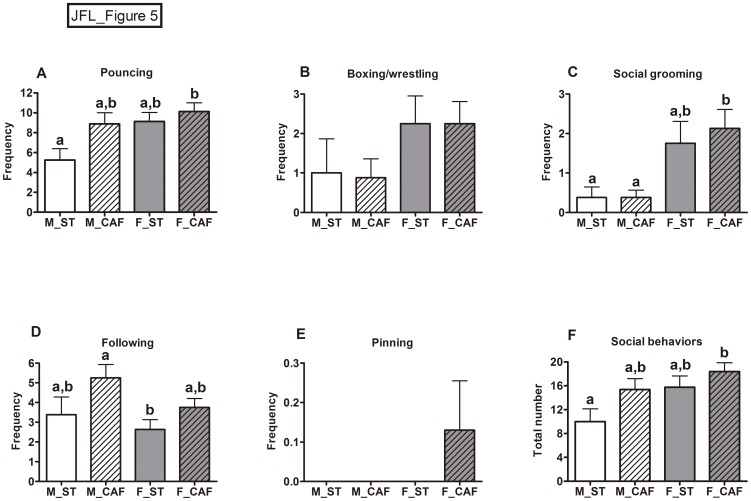
Social play test. The data represent the mean ± SEM of the following behaviours (n = 8): A) pouncing (jumping on or attacking the partner's nape); B) boxing/wrestling (rats stand on their hind paws and struggle using the forepaws); C) social grooming (sniffing or licking the partner); D) following/chasing (pursuing the partner); E) pinning (holding the partner in a supine position); and F) the total number of social play behaviours. Male (M) and female (F) rats were fed from weaning (at day 21 of life) and for 8 weeks with standard chow (ST) or the cafeteria diet (CAF). Two-way ANOVAs indicated that the cafeteria diet increased the frequency of pounces and pursuits and the total number of social behaviours (diet effect in all three variables *p*<0.05). Females showed more pouncing, grooming and total number of behaviours than males (two-way ANOVA, gender: *p*<0.05), as well as a residual increase in boxing (two-way ANOVA, p = 0.059). Males slightly increased the number of pursuits in comparison with females (two-way ANOVA, *p* = 0.098). ^ab^Mean values with unlike letters were significantly different between groups (one-way ANOVA and Tukey's *post hoc* comparison, *p*<0.05).

CAF-fed animals increased the frequency of social behaviour and specifically increased the frequency of pouncing and following (Figures 5A, 5D and 5F). The patterns of social interaction differed between genders. Females did more following, social grooming and total interactions than males (Figures 5A, 5C and 5F), and also showed a residual increase of boxing/wrestling episodes compared with males (Figure 5B). Males slightly increased the number of pursuits in comparison with females (Figure 5D).

### Effects Of Caf Withdrawal On Body And Tissue Relative Weights, Biochemical Parameters And Behaviour

The group of males fed the CAF diet for 7 weeks and the ST chow only during the 8th week (CAF withdrawal) showed a slightly reduced body weight gain in comparison with the CAF-fed group of males, and had become overweight by only 8.6% compared to the ST-fed group (Table 1 and Figure 1), which is qualitatively less than the 16.3% overweight shown by CAF-fed male rats. Moreover, CAF withdrawal reversed the relative liver weight (%) increase observed in the males of the CAF-fed group (Table 1).

CAF withdrawal and the shift to ST feeding only during the 8th week restored insulin sensitivity and completely reversed the increase in glucose, insulin and triglyceride plasma levels induced by CAF feeding during the previous 7 weeks (Table 1).

The analysis of exploration in the hole board (HB), as well as the data for the EPM and the social interaction test revealed no significant changes in the CAF withdrawal group compared to the CAF-fed animals (data not shown). However, analysis of the time spent in the centre of the open field showed a significant effect (one-way ANOVA: F(2,22) = 6.33, *p*<0.01; means ± SEM: M_ST, 57.74± 9.02; M_CAF, 98.73± 9.59; M_ABS, 86.27± 5.7). *Post hoc* comparisons revealed that CAF withdrawal in the last week partially restored the differences, as the CAF-fed group spent significantly more time in the centre of the open field compared to the ST-fed group (*p*<0.01, Tukey's test), whereas the ST-fed and CAF withdrawal groups showed a residual difference (*p* = 0.073). This result indicated that the administration of standard chow during the last week partially reversed the anxiolytic effect produced by CAF feeding. No significant effects were found for the distance travelled.

## Discussion

The present study shows that a palatable CAF diet administered from weaning to male and female rats increased food and fluid consumption, induced body weight gain, increased adiposity and liver weight, and produced different metabolic disturbances strongly related with the development of the metabolic syndrome, such as hypertriglyceridemia, hyperglycaemia and insulin resistance. It is known that CAF feeding induces hyperphagia, increases food, energy, fat and carbohydrate intake [23] leading to overconsumption of the high energy density foods contained in the diet [23]. Moreover, the deleterious effects of the CAF diet on body weight, adiposity, plasma lipid profile and insulin sensitivity have been well described [5]–[7],[24]. The response to CAF diet was different between males and females in some parameters, such as RWAT weight and cholesterol that increased more in CAF-fed males than in CAF-fed females and triglycerides that increased more in CAF-fed males than in CAF-fed females. These results are in line with the reported sex-associated differences in the regulation of proteins controlling body weight [25], and with the gender differential metabolic responses to the consumption of a palatable diet found after administering distinct forms of early stress to the animals [26],[27].

Behavioural results indicated that CAF-fed animals were less active than ST-fed animals in all behavioural tests. They consistently crossed a smaller number of squares in the hole board and travelled shorter distances in the enclosed arms of the EPM and in the open field, in comparison with the ST-fed controls. This behavioural pattern concurs with the decreased activity (and hyperphagia) shown by a knockout obese rat model for monogenic obesity in humans [28], and by obese adult rats fed with a high-fat diet [19]. In contrast, Souza et al [29] reported no differences in open field crossings (but increased anxiety-like behaviour) after four months of palatable diet feeding, compared with standard chow feeding. Curiously, no body weight differences were detected as a result of the diet in that study, even though body fat mass, insulin sensitivity and glucose tolerance were all altered in animals fed the palatable diet. Moreover, no changes in locomotor activity or body weight were reported after one week of a choice diet containing chow, lard and sucrose, which increased blood glucose and fat stores (mesenteric, epididymal, inguinal and perirenal white adipose tissues), or after 4 weeks of high fat, high carbohydrate chow [30],[31]. In humans, a longitudinal study performed with twin pairs discordant for obesity in young adulthood reported that obese co-twins were less active than non-obese co-twins according to an interview and questionnaire indices, and confirmed by accelerometers [32]. These results corroborate that obesity contributes to reducing activity. Hence, taken altogether, these results suggest that the overweight or obesity induced by the diet appears to be a specific and critical requirement for producing a significant decrease in activity levels.

A relevant finding of our study is that, for almost all variables analysed, the CAF diet produced an increase in exploratory behaviour, as well as a robust anti-anxiety effect. The CAF diet specifically increased head-dipping behaviour in the hole board, which has been validated as a measure of exploration [21]. Interestingly, selectively bred low-anxiety rats also showed increased head-dipping behaviour [33]. Moreover, manipulations that reduce anxiety, such as environmental enrichment [34], also increased exploratory activity in the hole board [35]. The results obtained in the present study consistently indicated that CAF feeding reduced anxiety. CAF-fed animals spent more time in the centre of the open field and increased the percentage of time and entries into the open arms of the EPM, although the magnitude of the effect varied by gender. In the open field, the effect was more pronounced in males than females, since males spent around 100 s in the centre during the 5-min test, whereas females spent 63 s. In the elevated plus maze the behaviour in the open arms was significantly enhanced to 35% in CAF-fed females compared to 20% in males. As rodents are fearful of open, unprotected or light spaces, the animals showing increased behaviour in such areas were interpreted as being less anxious [22],[36]. Previous studies have consistently reported that a palatable CAF diet reduces the negative impact of early stress in terms of anxiety, depression-like symptoms and hypothalamic-pituitary-adrenal (HPA) activity [29],[37]. There is a discrepancy with one study showing increased anxiety and basal corticosterone levels, but partly increased social interaction in obese rats fed with a high-fat diet [19]. Inconsistent results could be due to methodological differences related to the type of rats, housing conditions or the testing procedure. However, it cannot be ruled out that reduced anxiety is a specific effect produced by palatable food, but not by other non-palatable obesity-inducing diets.

In agreement with the low-anxiety behavioural profile shown in the other tests, animals fed with the CAF diet also showed increased social behaviour and, more specifically, they did more pounces and pursuits than ST-fed rats. Social behaviour has been validated and used for detecting anxiolytic drugs [38],[39] and, as a rule, increases in social behaviours indicate low anxiety levels. The main social interaction behaviours in adult rats for which scores have been recorded are social grooming (sniffing and licking), following, boxing and wrestling [38]. In the present study, scores were also recorded for pouncing and pinning, which are social behaviours related with social play in younger rats. Pouncing is considered to be the initial behaviour for soliciting play, while pinning is the most characteristic posture in the social play of juvenile rats [40]. Social play has stress-reducing effects, is rewarding and is crucial for the development of social skills and competences [40]. Our assumption was that, as our animals were young adults aged 10–11 weeks at the time of testing, some play behaviour would be observed. Therefore, the fact that CAF feeding increased pouncing (but not pinning) as well as the total number of social behaviours indicated that the CAF diet enhanced social interest and motivation to play. Moreover, this is consistent with the low levels of anxiety observed in the other tests of the present experiment, with those stress-reducing effects that have already been attributed to palatable food [41], and with another study reporting a reduction of the stress hormone response and a decrease in the corticotropin-releasing factor mRNA levels in the hypothalamus of animals fed with comfort food [42].

We do not know which mechanisms underlie the anxiolytic action of the CAF diet. Several systems modulating anxiety and related with food and feeding behaviour could be candidates. One is the CRF-CRF1 receptor system of the nuclei of the amygdala that has been recently involved in excessive eating of palatable food [43]. A second candidate is the type 1 cannabinoid (CB1) receptor that renders food more or less pleasurable [44] and mediates the reduction of anxiety-like behaviour induced by the inhibition of the endocannabinoid-degrading enzyme fatty acid amide hydrolase (FAAH) [45]. Another potential system is the benzodiazepines (BZs)/GABAA receptor complex, which contains the site of action of many anxiolytics, anticonvulsants and hypnotics, and mediates benzodiazepines-induced hyperphagia [46]. Further research about any of these systems will help to increase clarification of the molecular mechanisms and the brain regions underlying the behavioural effects of the CAF diet.

Yet, it has also been reported that post-weaning isolation rearing increased anxiety-like behaviour, decreased social interaction and altered hormonal response to stress in both rats [47] and mice [48]. Furthermore, it has been shown that chronic stress can increase the ingestion of palatable food [42]. Hence, it cannot be ruled out that the negative mood associated to isolation rearing contributed to increase food intake, weight gain and obesity in the present animals, which were also isolated after weaning. However, in a different experiment performed in our laboratory with pair-housed rats, body weight gain curves were similar to those found in the present study by both CAF- and ST-fed rats (data not shown). In an exhaustive review, Fone and Pakess [49] argued that the full behavioural change associated with social isolation in rodents requires a strict protocol, and that any form of contact with a conspecific or excessive handling by the experimenter can negate the expected isolation-induced changes. As the CAF diet required daily fresh food, CAF-fed animals were handled daily when measuring water and chow consumption, as were the ST-fed animals. Those daily interventions involved some interaction with the animal and decreased the level of isolation. Further experiments, which allow direct comparisons to be made between isolated and group-housed rats fed with either CAF or ST diets, are required to confirm the possibility that daily interventions reduced the impact of rearing in complete isolation, and increased the “dampening effect” on the final amount of stress suffered by the animals.

Surprisingly, CAF withdrawal during the final 8th week allowed the animals to recover triglycerides, glucose and insulin sensitivity, as well as liver weight. Some studies have shown an improvement in different parameters clustered in the metabolic syndrome in rats switched from the CAF diet to the ST diet [50]–[52]. In a study performed with adult male Sprague-Dawley rats fed with a CAF diet for 16 weeks, the switch from the CAF diet to ST chow for 9 days produced a significant decrease of the white adipose tissue mass and the plasma levels of leptin and glucose [51]. Ong et al [52] found similar results in female, but not in male, Albino Wistar rats fed with a CAF diet for 8 weeks and then switched to chow for 72 hours. However, in any of these studies no changes were found in plasma insulin levels and no data related to the plasma levels of triglycerides, liver weight and insulin sensitivity were available [51],[52]. Therefore, to the best of our knowledge, the present study provides new evidence of a beneficial recovery effect on many parameters clustered in the metabolic syndrome, after only one week of CAF withdrawal.

The animals withdrawn from the CAF diet behaved like CAF-fed animals in most of the behavioural tests except in the open field, where they showed a decrease in the time spent in the centre, thereby indicating increased anxiety compared to the CAF-fed animals. This is consistent with an interesting study of Pickering et al [53] who also found increased anxiety in the open field in diet-induced obese rats after 2 weeks of diet withdrawal. Animals receiving a high-fat high-sugar (HFHS) diet for 7 weeks were divided into obesity-prone and obesity-resistant groups based on the relative weight gain and tested 2 weeks after withdrawal. Interestingly, the withdrawal from the HFHS diet only induced craving in obesity-prone animals, but not in obesity-resistant animals also fed with the HFHS diet, thus suggesting that negative emotions associated with food withdrawal could depend on the severity of overweight or obesity.

In conclusion, the present study shows that CAF feeding of young animals robustly yields reduced activity and decreased anxiety in male and female rats not suffering stressful circumstances in either their past or present life. A major new finding is the partial recovery from some metabolic alterations found only one week after CAF withdrawal, which also occurred simultaneously with an increase of anxiety levels. In addition, our study indicates the behavioural and metabolic implications of CAF feeding in young animals, and that further research is needed to evaluate possible recovery after withdrawal.

## Methods

### Ethics Statement

The experimental protocol was approved by the *Generalitat de Catalunya (DAAM 6643)*, following the ‘Principles of laboratory animal care’ and was carried out in accordance to the European Communities Council Directive (86/609/EEC).

### Animals And Housing Conditions

The animals were male and female Sprague-Dawley rats bred and raised at the main animal facility of the *Universitat de Barcelona*. They were weaned at 21–22 days of age, housed singly, maintained in standard conditions of temperature (22°C±2°C), humidity (50±10) and on a 12–12 h light-dark schedule (lights on at 0800h), and fed with the corresponding diet (see below).

### Cafeteria And Standard Diets

The rats were fed from weaning (at day 21 of life) and for 8 weeks with either a standard chow (ST) (Harlan, Barcelona, Spain) (M_ST and F_ST groups) or a CAF diet (M_CAF and F_CAF groups) with the following components (quantity per rat): bacon or frankfurter (8–12 g); biscuit with pâté (12–15 g); biscuit with cheese (10–12 g); muffins or *ensaimada* (pastry) (8–10 g); carrots (6–8 g); milk with sugar (220 g/l; 50 ml); water (*ad libitum*); and ST chow. The ST chow had a calorie breakdown of 24% protein, 18% fat and 58% carbohydrates, whereas the calorie breakdown of the CAF diet was: 10% proteins; 41% fat; and 49% carbohydrates. It is worth noting that not all the components of the CAF diet are equally eaten by the animals, as they are accustomed to having a higher preference for compounds that have a higher lipid content [7]. The animals were fed *ad libitum*, and the food was renewed daily. Body weight was monitored weekly over 8 weeks. A third group of males was fed with the CAF diet for 7 weeks and with ST chow only for the 8th week (M-ABS group) to determine the effects of CAF diet withdrawal during the last week of the study.

Food was withdrawn 12–14 h before sacrifice. Animals were sacrificed by beheading and total blood collected. Serum was obtained by centrifugation at 4°C, 2000 g for 15 minutes and stored at −80°C until further use. Retroperitoneal white adipose tissue (RWAT) and liver were dissected and weighed.

### Plasma Biochemical Analysis

Enzymatic colorimetric kits were used for the determination of serum glucose, triglycerides, cholesterol (QCA, Barcelona, Spain) and free fatty acids (WAKO, Neuss, Germany). Serum insulin was determined by a rat/mouse ELISA kit (Millipore, Barcelona, Spain) following the manufacturer's instructions.

### Homa-Ir Analysis

Homeostasis model assessment-estimated insulin resistance (HOMA-IR) was calculated following the formula: (Glucose X Insulin)/22.5 as described previously [54].

### Behavioural Procedures

Behavioural experiments were carried out during the 7^th^ and 8^th^ weeks of administering the diets, when the animals were 10 and 11 weeks of age.

Animals were tested for exploratory activity in the hole board (HB) (day 1), anxiety in the elevated plus maze (EPM) (day 2), locomotion and activity in the open field (day 3) and social interaction (day 5). All experiments were carried out in the morning (between 9:00 am and 2:00 pm) in an isolated experimental room with dim lighting. All procedures were videotaped and further analysed by a researcher who was unaware of the treatment that each animal had received.

### Hole-Board Test

The hole-board test for measuring exploratory behaviour [20] consisted of a white (66×66×47 cm) wooden box with four equidistant holes (3.7 cm in diameter, 18 cm deep) in the floor, which was divided into 16 equal squares with a red marker. The animal was placed in the centre of the floor apparatus and allowed to explore the holes for five minutes. The number of squares crossed (ambulation), the number of head dips and the time spent head dipping were measured.

### Elevated Plus Maze Test

The EPM test [21] apparatus consisted of four arms (44 cm long × 10 cm wide) made of black formica extending from a 10 cm square centre positioned 90° from each other to form the shape of a plus sign. Two of the opposing arms had wooden walls (closed arms, 40 cm high), whereas the other two were the open arms that had only a 0.5 cm ridge to provide additional grip. The whole maze was elevated 50 cm above the floor. The rat was placed in the centre of the maze (always facing the same closed arm) and, for five minutes, scores were recorded for the number of entries into the open and closed arms (defined as placing all four paws into a given arm), and for the total time spent in the open and closed arms. The distance travelled in the enclosed arms was video tracked (Smart 2.4, Panlab, Barcelona, Spain). The behaviour in the open arms is indicative of the anxiety levels of the animal, with a high amount of time and entries into the open arms being indicative of low anxiety.

### Open Field Test

The open field was a wooden grey box (75×45×45 cm). A central area (20 cm × 40 cm) was draw in the floor of the apparatus to score the time in this central zone that is considered to indicate decreased anxiety [55]. The animals were individually placed in and allowed to explore the apparatus for 5 min. The distance travelled and the time spent in the central area were video tracked.

### Social Interaction Test

The social play interaction test was performed in the open field where the animals had been previously tested. The animals were again placed in the open field the day before the social interaction test to allow habituation to the apparatus [39]. Naive juvenile male and female rats (5-week-old) were used as social stimulus for interaction. The test consisted of placing the experimental rat and a juvenile rat of the same sex in the box for 5 min. Trials were videotaped and a trained observer, who was blind to the animal's treatment, scored the following social behaviours [40]: pouncing (jumping on or attacking the partner's nape, which is considered to be an initiation or solicitation of play); boxing/wrestling (both rats stand on their hind paws and struggle using the forepaws; usually boxing follows pouncing and is considered to be a positive partner's response to play); following/chasing (moving in the direction of or pursuing the partner); social grooming (sniffing or licking any body part of the test partner) and pinning (the rat holds the partner in a supine position).

### Statistical Analysis

The data were analysed using the “Statistical Package for Social Sciences” (SPSS, version 17.0) using ANOVA. Two analyses were undertaken. The first one evaluated the effects of gender and diet, as well as the interaction between these factors (two-way ANOVA; gender (male, female) × diet (ST, CAF)); four groups were included (M_ST, M_CAF, F_ST and F_CAF). The second analysis evaluated the effects on males of the CAF diet administered for 8 weeks, and the effects of the CAF diet for 7 weeks and ST chow feeding during the final week (one-way ANOVA); three groups were included (M_ST, M_CAF and M_ABS). *Post hoc* comparisons between groups were performed by Tukey's multiple range tests after significant ANOVA. Differences between diets were analysed using the independent Student's t-test across groups. Body weight data were analysed by a repeated measures ANOVA, with week as a within-subject factor (among 9 measurements, including initial body weight at weaning).

## Supporting Information

Table S1 **
Average daily intake (mean ± SEM) of standard chow, fluids, nutrients and energy over the 8 weeks of the experiment.** Male (M) and female (F) rats were fed from weaning (at day 21 of life) and during 8 weeks with a ST (M_ST and F_ST groups) or a CAF (M_CAF and F_CAF groups) diet. An extra group of males were fed with the CAF diet during 7 weeks and with only the ST diet the last week of the study (M_ABS group). g = grams, d = day.
(DOCX)
