# Supplementary material for: Effects Of A Post-Weaning Cafeteria Diet In Young Rats: Metabolic Syndrome, Reduced Activity And Low Anxiety-Like Behaviour
Source: PLoS One. 2014 Jan 15;9(1):e85049. doi: 10.1371/journal.pone.0085049 (PMC3895047; doi:10.1371/journal.pone.0085049)
Supplement: Table S1 — Average daily intake (mean ± SEM) of standard chow, fluids, nutrients and energy over the 8 weeks of the experiment. Male (M) and female (F) rats were fed from weaning (at day 21 of life) and during 8 weeks with a ST (M_ST and F_ST groups) or a CAF (M_CAF and F_CAF groups) diet. An extra group of males were fed with the CAF diet during 7 weeks and with only the ST diet the last week of the study (M_ABS group). g = grams, d = day. (DOCX) [file pone.0085049.s001.docx]

**Supplementary Table. Average daily intake (mean ± SEM) of standard chow, fluids, nutrients and energy over the 8 weeks of the experiment.**

|  | **M_ST** | **F_ST** | **M_CAF** | **M_ABS** | **F_CAF** |
| --- | --- | --- | --- | --- | --- |
| **Chow (g/d)** | 20.0 ± 0.3 | 16.0 ± 0.3 | 7.3 ± 0.6 | 9.3 ± 0.5 | 4.6 ± 0.2 |
| **Total solid food (g/d)** | 20.0 ± 0.3 | 16.0 ± 0.3 | 26.6 ± 0.6 | 28.5 ± 0.7 | 21.1 ± 0.8 |
| **Water (g/d)** | 28.9 ± 0.8 | 27.6 ± 1.1 | 13.8 ± 1.1 | 16.1 ± 0.8 | 11.7 ± 0.7 |
| **Total fluid (g/d)** | 28.9 ± 0.8 | 27.6 ± 1.1 | 62.0 ± 2.1 | 57.5 ± 2.6 | 61.7 ± 2.2 |
| **KCAL** | 68.0 ± 0.9 | 54.5 ± 0.9 | 127.4 ± 3.0 | 124.8 ± 3.3 | 116.5 ± 2.5 |
| **Protein** | 3.8 ± 0.0 | 3.0 ±0.1 | 6.3 ± 0.1 | 6.2 ± 0.1 | 6.2 ± 0.0 |
| **Fat** | 1.2 ± 0.0 | 1.0 ± 0.0 | 3.9 ± 0.1 | 3.9 ± 0.1 | 3.5 ± 0.1 |
| **Carbohydrate** | 10.0 ± 0.1 | 8.0 ± 0.1 | 18.8 ± 0.5 | 18.0 ± 0.6 | 17.6 ± 0.5 |
| **Fibre** | 0.8 ± 0.0 | 0.6 ± 0.0 | 0.6 ± 0.0 | 0.7 ± 0.0 | 0.5 ± 0.0 |

Male (M) and female (F) rats were fed from weaning (at day 21 of life) and during 8 weeks with a ST (M_ST and F_ST groups) or a CAF (M_CAF and F_CAF groups) diet. An extra group of males were fed with the CAF diet during 7 weeks and with only the ST diet the last week of the study (M_ABS group). g=grams, d=day.
